# Tactile Interaction Sensor with Millimeter Sensing Acuity

**DOI:** 10.3390/s21134274

**Published:** 2021-06-22

**Authors:** Eunsuk Choi, Sunjin Kim, Jinsil Gong, Hyeonjeong Sun, Minjin Kwon, Hojun Seo, Onejae Sul, Seung-Beck Lee

**Affiliations:** 1 Department of Electronic Engineering, Hanyang University, 222 Wangsimni-ro, Seongdong-gu, Seoul 04763, Korea; silver77@hanyang.ac.kr (E.C.); akangel0307@gmail.com (S.K.); jytotop@hanyang.ac.kr (J.G.); try1717@naver.com (H.S.); youngforever0213@naver.com (M.K.); masiks@hanyang.ac.kr (H.S.); 2 Institute of Nano Science and Technology, Hanyang University, 222 Wangsimni-ro, Seongdong-gu, Seoul 04763, Korea; ojsul@hanyang.ac.kr

**Keywords:** digitized output, tactile sensor, pressure sensor, shear sensor

## Abstract

In this article we report on a 3 × 3 mm tactile interaction sensor that is able to simultaneously detect pressure level, pressure distribution, and shear force direction. The sensor consists of multiple mechanical switches under a conducting diaphragm. An external stimulus is measured by the deflection of the diaphragm and the arrangement of mechanical switches, resulting in low noise, high reliability, and high uniformity. Our sensor is able to detect tactile forces as small as ~50 mgf along with the direction of the shear force. It also distinguishes whether there is a normal pressure during slip motion. We also succeed in detecting the contact shape and the contact motion, demonstrating potential applications in robotics and remote input interfaces. Since our sensor has a simple structure and its function depends only on sensor dimensions, not on an active sensing material, in comparison with previous tactile sensors, our sensor shows high uniformity and reliability for an array-type integration.

## 1. Introduction

The tactile sense of human fingers plays an essential role in object manipulation and interaction with a contacting object. When we make contact with an object, we can recognize its texture and shape through the mechanoreceptors distributed under the skin [1]. We can also grip and manipulate objects using appropriate grip forces from monitoring the slip motion of the object [2]. Various tactile sensors have been developed for robotics and biomedical applications that have the ability to detect pressure magnitude, pressure distribution, and slip, similar to the human tactile senses [3,4,5]. The previously reported tactile sensors mainly focus on pressure detection, and they have demonstrated extremely high pressure sensitivity, enough to detect insects landing [6,7,8] or to recognize object shapes using a sensor array [9,10,11]. However, for robotics and biomedical applications, shear detection is required as well as the detection of pressure and its distribution for object identification or object manipulation without slip [12]. Many sensors developed so far have simultaneous sensing abilities on vertical and shear forces, but they do not discriminate them [13,14,15]. To resolve this issue, integrated sensor systems have been developed to distinguish the pressure and shear stress, utilizing mechanisms such as differential capacitance depending on force directions [16,17,18], a bump structure discriminating torsional or non-torsional strain [19,20,21], or piezo-resistive response difference determined by cantilever or beam deflection direction [22,23].

The tactile sensors with high sensitivity are based on nanomaterials, for example, carbon nanotubes or graphene as active sensing materials [24,25,26]. These nanomaterials showed high resistance change ratios on external stimulus; however, they lack applicability due to issues of non-uniformity and non-reproducibility [27]. In addition, in the case of capacitor type sensors, there were cross-talk and noise issues for measuring low magnitude stimulus at high spatial resolution [28]. They are prone to environmental charge and have structural complexity. Thus, reliability against noise and structural simplicity are required.

In this report, we introduce a tactile interaction sensor (TIS) that can simultaneously detect contact pressure, contact shape, and shear direction. The TIS sensor consists of pressure sensors and shear sensors based on mechanical switches under diaphragm deflection. Under the diaphragm, there are multiple switches so that a larger stimulus results in a larger contact area between the diaphragm and the bottom substrate and a larger number of shorted switches, producing larger output current levels. Mechanical switches have intrinsically large signal-to-noise ratio and high reliability, so are thus less susceptible to environment issues. We integrated multiple pressure sensor and shear sensor units on the TIS within a 3 × 3 mm area. The TIS is also effective in the recognition of a contacting material’s shape and its movement in real time.

## 2. Device Design and Operating Mechanism

**Figure 1:**
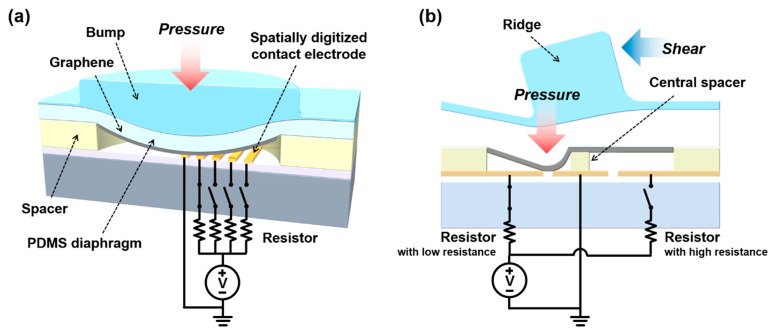
The schematic illustration of a pressure sensor unit and a shear sensor unit integrated on the TIS: (**a**) 3D cross-sectional schematic diagram showing the operating mechanism of the pressure sensor unit; (**b**) cross-sectional schematic diagram showing the shear detecting mechanism of the shear sensor unit.

This TIS was designed based on our previous reports utilizing mechanical switches under diaphragm deflection [29,30]. To simultaneously detect shear direction and pressure distribution at millimeter scales, we integrated four pressure sensor units and three shear sensor units on TIS within a 3 × 3 mm area. Figure 1a shows a schematic diagram of the unit structure of the pressure sensor based on a mechanical switch under diaphragm deflection. The top layer is a PDMS diaphragm, and graphene is at the bottom surface of the diaphragm as a flexible electrode. The bottom structure consists of a spacer pit, a ground electrode, and spatially digitized contact electrodes (CEs) with individually associated resistors. As seen in Figure 1a, when vertical pressure is applied to the sensor, the PDMS diaphragm is deflected into the pit. Depending on the pressure, the number of CEs shorted with the ground electrode is determined, and a proportional current level is generated. In comparison to our previous sensor [29], the base sensor structure having a conducting diaphragm and CEs is similar. However, in this study we utilized graphene, not metallic thin-film, as the electrode under the diaphragm to improve the operating mechanical reliability on repetitive diaphragm deflection. If a metal electrode is used instead of graphene, the repetitive deflection of the diaphragm can generate cracks, reducing sensor reliability.

Our pressure sensor function depends only on the number of the conducting paths, not on the active sensing material. Therefore, our sensor has a large signal-to-noise ratio, is reliable, and is less susceptible to environmental changes. Our sensor can also be tailored in terms of sensing range and sensitivity. According to diaphragm deflection theory [31], diaphragm deflection magnitude depends on the pit diameter, diaphragm thickness, and diaphragm modulus. The diaphragm deflection magnitude and the gaps between the ground electrode and the CEs determine threshold pressures for generating conducting paths. Therefore, the sensitivity and the sensing range can be tailored by controlling the diaphragm parameters and the CEs arrangement.

The shear sensor has a similar structure to the pressure sensor; however, a central spacer and a ridge structure are added (Figure 1b). Shear forces applied to the ridge structure generate a torque around the central spacer, which acts as the axis of rotation, and the torque is converted to a vertical pressure, creating diaphragm deflection. Two opposing sensing elements that are separated by the central spacer are connected with their associate resistors with different resistances. Thus, the shear direction can be detected from different current levels. In this study, to increase shear transfer characteristics, we used a PDMS layer with low modulus and an SU-8 ridge structure with high modulus [32].

## 3. Device Fabrication

### Fabrication Process

**Figure 2:**
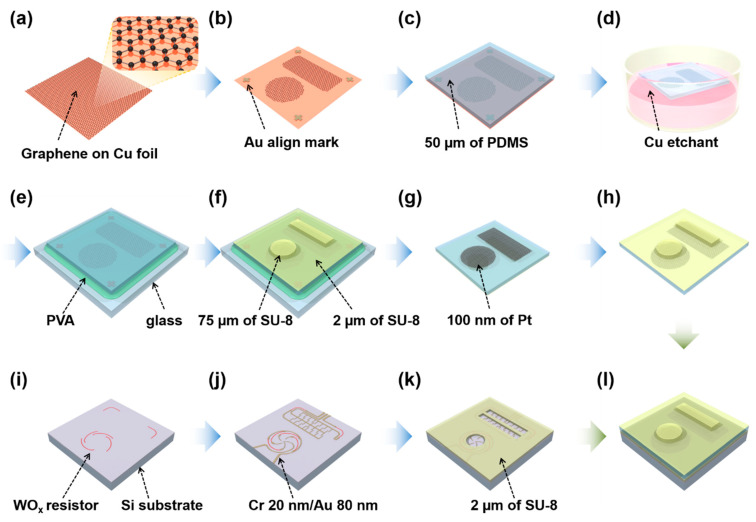
The fabrication process of TIS: (**a**) graphene grown on Cu foil; (**b**) align mark deposition and graphene patterning; (**c**) PDMS molding; (**d**) Cu foil etching; (**e**) fixing PDMS layer to glass substrate using PVA; (**f**) forming bump and ridge structure; (**g**) Pt deposition; (**h**) fabricated top layer; (**i**) WO_x_ resistor formation; (**j**) electrode deposition; (**k**) spacer formation; (**l**) combining with fabricated top layer and bottom substrate.

The fabrication processes of the TIS can be divided into two parts: one for the top structure (Figure 2a–h) and the other for the bottom structure (Figure 2i–k). First, the graphene grown on Cu foil (Graphene Square Inc., Seoul, Korea) was attached on a PET film, and Au align marks for the subsequent lithography process were formed on graphene/Cu foil using thermal evaporation with a stencil mask. The graphene on Cu foil was patterned by an O_2_ plasma etching process. Then, the PDMS base with mixed cross-linker was poured on the patterned graphene and cured. The volume of the poured PDMS was controlled to form a 50-μm-thick PDMS. After etching the Cu foil using a copper etchant, the patterned graphene was transferred to the PDMS layer [33,34]. Commonly, graphene is patterned after the wet transfer method using PMMA; however, in this study graphene was transferred to PDMS after patterning to prevent ripples and cracks in the graphene due to the chemical swelling effect of the PDMS layer during the development in the lithography process [35].

The PDMS layer with patterned graphene was fixed to a glass supporting substrate using water-soluble polyvinyl alcohol (PVA). PVA prevents the graphene from transferring to the glass supporting substrate during subsequent baking and lithography processes using SU-8 for fabricating the ridge and bump structures. PVA has high chemical resistance to SU-8 and the SU-8 developer. The top surface of the PDMS layer was treated using oxygen plasma for better adhesion with an SU-8 2002 thin (2 μm) film coating. This SU-8 2002 thin layer works as the adhesive layer between the PDMS and the following second SU-8 2075 layer. The 75 μm of the second SU-8 2075 layer was spin-coated, and SU-8 ridges and bumps were fabricated using optical lithography. Finally, the PVA sacrificial layer was removed using deionized water and a 100 nm Pt film was deposited following the shape of the patterned graphene. A Pt electrode was used for increasing the conductivity of the flexible graphene electrode, similar to a previously reported hybrid layer [36,37].

The bottom structure fabrication process begins with the defining of tungsten oxide (WO_x_) resistors on an Si substrate using optical lithography (Figure 2i). The WO_x_ resistors were fabricated by sputtering tungsten in oxygen atmosphere. After forming a 2-μm- thick SU-8 spacer, Cr/Au (20 nm/100 nm) electrodes were evaporated as contact electrodes. Finally, the fabricated top and bottom structures were combined.

**Figure 3:**
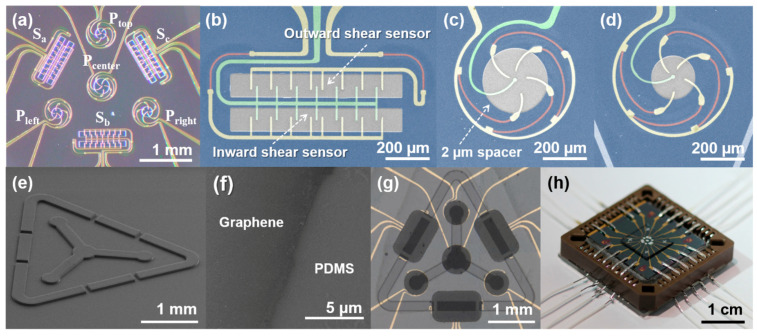
The image of the fabricated TIS: (**a**) an optical image of the bottom substrate with four pressure sensor units (P_center_, P_top_, P_left_, and P_right_) and three shear sensor units (S_a_, S_b_, an S_c_); the false color SEM image of (**b**) a shear sensor unit and pressure sensor units with (**c**) 200 μm and (**d**) 300 μm pit diameter. Each sensor is composed of (yellow) contact electrodes, (red) resistors, (green) ground electrode, and (blue) spacer; (**e**) SEM image of the SU-8 ridge and bump structure on the top PDMS layer; (**f**) SEM image of the patterned graphene after transferring to PDMS; (**g**) an optical image of the TIS sensor after combining the top and bottom substrates; (**h**) the fabricated TIS after mounting in a chipset.

Figure 3a shows an optical image of the fabricated bottom structure of TIS. The TIS is composed of a center pressure sensor (P_center_) with higher sensitivity than that of the three outer pressure sensors (P_top_, P_left_, and P_right_), and the three shear sensors (S_a_, S_b_, and S_c_). All of the pressure and shear sensors are located inside a 3 × 3 mm area on a single chip. As seen in Figure 3b, the shear sensor consisted of two 120 × 900 μm pits and a 60-μm-wide central spacer, which acts to divide the outward sensor and the inward sensor. The contact electrodes (yellow) and the ground electrode (green) were designed as a comb structure for detecting the shear in any region of the shear sensor.

The pit diameter of the pressure sensor with higher sensitivity (Figure 3c) and lower sensitivity (Figure 3d) were designed to be 300 and 200 μm, respectively. The gaps between CEs are 15 μm. The CEs in the pressure sensor are arranged in a spiral to reduce the overall size of the individual sensor within a 0.3 mm^2^ area. For the mechanical and chemical stability of WO_x_ resistors, the resistors are placed under the SU-8 spacer. The resistance of the resistors are 39 kΩ (red) and a 2-μm-thick SU-8 spacer is used for all of the pressure and shear sensors. In the case of the shear sensor, a 33 kΩ additional resistor is connected with the outward sensor, and the inward sensor and the outward sensor are connected in parallel, enabling discrimination between the outward and inward shear stresses by measuring the output signals of the shear sensors.

Figure 3e shows the fabricated top structure of the TIS. The top structure is composed of a 50-μm-thick PDMS, and the thicknesses of the SU-8 bump and the ridge structure are 75 μm. The diameters of the bump structure in the center pressure sensor and the outer pressure sensor were fabricated to be 400 and 300 μm, respectively. The bump structure improves the pressure sensitivity by localizing the contact stimulus to the pit area [38,39]. As seen in Figure 3f, the clearly patterned graphene with no ripple and crack was transferred to the underside of the PDMS layer. The fabricated top and bottom structures were aligned and attached (Figure 3g). Finally, the fabricated TIS was loaded on a chip mount for operation, as shown in Figure 3h.

## 4. Operating Characteristics

**Figure 4:**
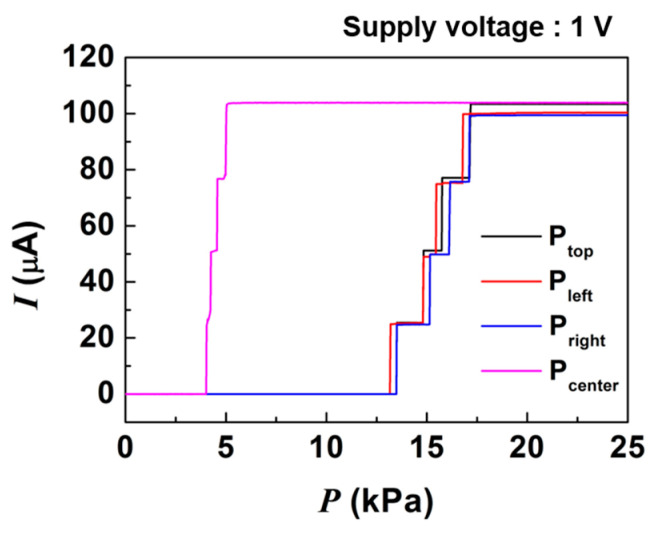
The pressure-dependent output characteristics of pressure sensor units.

Figure 4 shows the responses of the four pressure sensors under 1 V bias and uniform pressure application over the entire device area using a 1 × 1 cm pressure tool and a motorized stage with a pressure sensor. The pressure sensors showed fully digitized output characteristics with five current steps of 25 μA depending on the applied pressure. P_center_ produced an earlier response due to its lower pressure threshold by the larger pit diameter, and the rest of pressure sensors (P_top_, P_left_, and P_right_) showed higher thresholds, as designed. The P_center_ had a 4 kPa threshold pressure, which corresponds to ~0.5 mN or ~50 mgf. When 100 kPa of pressure, which is above the sensing range of the pressure sensor, was applied to a graphene layer, the resistance change was estimated to be about 0.59% [40]. Therefore, we assumed that the measured results were not affected by any fluctuation in the graphene resistance. In the pressure sensors (P_top_, P_left_, and P_right_), only 3% current variation was observed, which may be due to misalignment during the lithography processes. This variation can be compensated by using a lithography process with high resolution or a software-based calibration technique.

In our previous work, our sensor structure showed high stability in the output current magnitude due to the reliable contact switching between the top electrode and CEs [29]. Thus, the pressure sensors possess output reliability and uniformity in the designed pressure sensing range.

In this study, our pressure sensor units showed a non-linear relationship between pressure and current. This non-linear relationship was caused by the non-linearly deflecting diaphragm, depending on linearly applied pressure, making contact with uniformly spaced CEs. Each pressure level showing stepped output could be tailored by controlling the distance between CE and the pit center. Therefore, by designing the CEs to have a smaller gap, the farther they are from the pit center, a linear output characteristic is produced; by designing a larger number of CEs and smaller gaps between CEs overall, a more continuous output characteristic can be attained [29].

**Figure 5:**
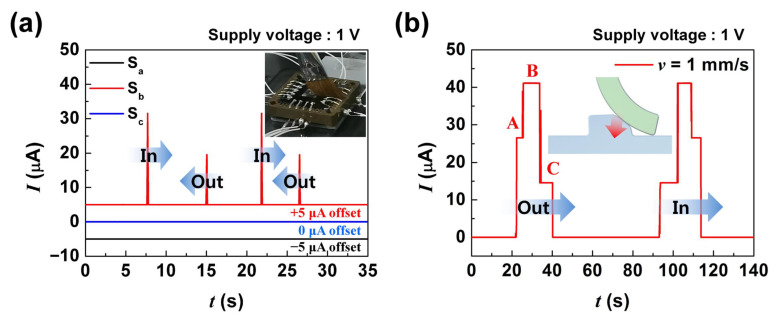
The shear-dependent output characteristics of the shear sensor units: (**a**) the measurement outputs of shear sensor units obtained from brush strokes shown in the inset; (**b**) the measurement output of shear sensor units obtained from PET strip scanning with vertical pressure.

Next, we tested the shear detecting characteristics of the shear sensor. The shear sensor was able to detect shear direction through the different current levels between inward and outward output currents. Figure 5a shows the responses of the three shear sensors when a paintbrush scanned, by hand, in both directions along the b-axis (from top to bottom in Figure 3a) without vertical pressure. In the case of sensor S_b_, 5 μA current was produced when the brush passed in the inward direction, whereas 14 μA was produced for the outward direction. Therefore, from the magnitudes of the current spikes, the shear direction could be determined. As shown, there was no cross-talk between S_b_ with S_a_ and S_c_ when the brush scanned along the b-axis directions.

Figure 5b shows the shear response of S_b_ when a PET strip was scanned in both directions along the b-axis with applied pressure and 1 mm/s scanning speed using a motorized stage. Unlike Figure 5a, the shape of the output signal shows three clearly distinctive current levels (A, B, and C in Figure 5b). In the case of pressurized slip, because the vertical pressure was dominant over the torque of the ridge, the inward shear sensor produced a current signal first, although the shear occurred along the outward direction (A phase). As the PET tip passed the ridge, the highest current level was observed due to the simultaneous operation of inward and outward shear sensors (B phase), and finally, only the outward sensor produced a current signal in the C phase. In the case of the inward direction scan, C phase appears first, and B and A phases follow sequentially. Thus, we concluded that our shear sensor can not only detect the direction of shear but also the presence of vertical pressures.

## 5. Shape and Motion Detection

**Figure 6:**
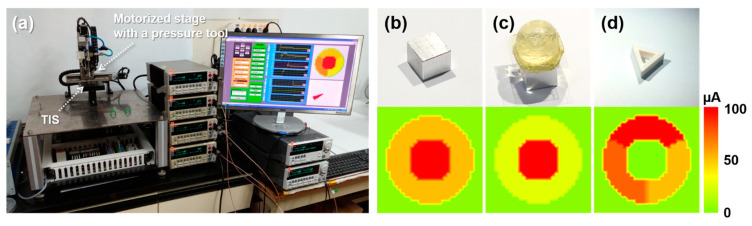
The output characteristics of the TIS during sensing operation: (**a**) the measurement set-up; the current contour map of pressure sensor units when (**b**) box, (**c**) sphere, and (**d**) hollow triangle objects pressed on the TIS.

TIS, which integrates four pressure sensors and three shear sensors, can recognize the shape and motion of contacting objects. For these measurements, we used the measurement setup shown in Figure 6a. The contact object was fixed to the motorized stage, and each sensor was connected with a source meter unit. The measurement program showed the measured current levels of pressure sensors as a color contour map, and the shear direction as a red arrow. Figure 6b–d shows the responses of the TIS under three different contacting objects: an aluminum box, a silicone sphere, and a 3D-printed hollow triangle smaller than a centimeter. Comparing the results of the sphere and the box, the sphere showed a lower current level of the outer pressure sensors than the box. This result verifies that our sensor discriminates between spherical and flat contact surfaces. In the measurement result of the hollow triangle, no current level in the center pressure sensor was observed to indicate that the contact object had a hollow structure. The current contour maps confirm that the distribution and magnitudes of the output signals provide information on the contact object shape. In this study, we designed the sensor dimension for approximately detecting contact shape with about 1 mm spatial resolution. Due to its structural advantage of having no dependency on active sensing materials, high spatial resolution for detailed shape detection can be easily attained while maintaining the uniform operating characteristics just by scaling sensor dimensions using a lithography process with higher resolution.

**Figure 7:**
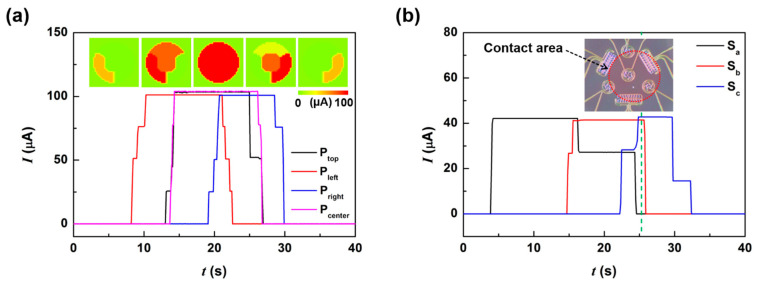
The output characteristic of the TIS depending on the sliding motion of a silicone sphere: (**a**) the measurement output of pressure sensor units, where the inset shows the variation in the current contour maps; (**b**) the measurement output of shear sensor units, where the inset shows the predicted contact shape at ~23 s (green dash line). These real-time measurement results are also shown in video S1.

Figure 7 shows the output signals of the TIS when the sphere in Figure 6c slid on the TIS from left to right with a 0.167 mm/s scanning speed. As shown in Figure 7a, the sequential pressure sensor output signals precisely corresponds to the position of the sphere. Figure 7b shows the output current of the three shear sensors measured simultaneously with the pressure sensors. We observed step-wise outputs from S_a_ and S_c_, similar to that shown in Figure 5b, and a single current step from S_b_. These results confirm that there was a pressurized slip along the right direction. The variations in each shear sensor produce temporal profiles of signals, hinting at the shape of the contacting object at each moment. For example, at the moment designated by the vertical dashed green line in Figure 7b, we can estimate the location and the elliptical shape (red circle) of the contacting object. During the sliding, we can assume that the elliptical contact shape is caused by the compressive deformation of the silicone sphere due to the surface friction. The shear direction can be detected from the current level change of many pressure sensors, as shown in Figure 7a; however, it is difficult to detect non-pressurized shear using only pressure sensors. For robotics and biomedical applications that require identification and manipulation of objects, shear sensors and pressure sensors must be integrated together. Video S1 and Figure S1 in the Supplementary Data includes the real-time output of the TIS during the measurements shown in Figure 7a,b. We also tested the detection characteristics on pressurized clockwise and counter-clockwise circular motions. We found that the TIS sensor can detect pressurized circular motion with fast response characteristics (Videos S2 and S3).

## 6. Discussion

The TIS has several advantages over previous tactile sensors in power consumption, reliability, and simplicity. TIS has low power consumption due to its structural feature composed on mechanical switches. In the absence of an external stimulus, the diaphragm does not make contact with the CE, and hence, there is no standby power loss at the sensor. In this study, our sensor showed ~33 dB of high signal-to-noise ratio (estimated from current measurements under constant stimulus). Therefore, it would be possible to further decrease the operating power consumption of the TIS by decreasing the supply voltage and increasing the resistance of the resistors connected with the CEs to a value where the resulting measurement noise does not exceed the step current distinguishing each detected pressure level.

The operating uniformity and reliability of a tactile sensor are essential factors required for forming a sensor array. In the cases of piezoresistive and capacitive tactile sensors, the operating uniformity and reliability of these types heavily depend on the electro-mechanical uniformity and reliability of the active sensing material. In particular, where novel nanomaterials with high sensitivity are used as the active sensing material, it is difficult to secure uniform electrical properties over a large area to form arrays of sensors. In comparison, the operating characteristics of our sensor using no active sensing material depend only on the designed sensor dimension. Therefore, the operating uniformity and reliability can be easily secured even if it is configured as an array. The TIS has a simple structure, fabrication process, and read-out circuit compared to MEMS-type tactile sensors. MEMS tactile sensors have been widely used in robotics and electronics owing to their high reliability; however, their fabrication process is complex and expensive. Especially, capacitive MEMS tactile sensors need complex readout circuits to prevent operational and environmental noise. However, our sensor’s simple structure allows it to be fabricated by a simple process at low cost, and our sensor’s high signal-to-noise ratio only needs a relatively simple readout circuit.

In our sensor structure, the number of sensing levels is determined by the number of arranged CEs, so it may be a limitation on applications that require readout of the timely display of numerical tactile pressure values. This limitation may be overcome by increasing the number of arranged CEs with closer spacing and reduced width. However, the ability to directly distinguish small spatial differences in tactile stimuli levels and the high reliability with high signal-to-noise ratio may allow the TIS to function as a stimuli magnitude level indicator for systems that utilize predesignated stimuli as highly reliable triggers for a desired action command.

The TIS is designed for detecting light touches in a 50~200 mgf range of contact pressure. This sensing range can be easily tailored by controlling the diaphragm dimension, the position of the CEs, and the number of the CEs for a particular application. The shear sensor of the TIS shows the ability to detect minimum pressure shear and its direction. In a typical biomedical application, recognition of shear stresses arising from contacting object slip would be desired rather than knowing the absolute magnitude of shear stress for material identification, thus triggering a motor response to increase grasp strength [12]. If necessary, the shear force magnitude can be detected through a simple process of increasing the number of CEs [30]. Since the TIS with a small active sensing area detects pressure level, pressure distribution, and shear forces direction, it may be possible to integrate it on a minimally invasive surgery robot [41], a prosthetic hand [42], and even a remote input interface that can be worn on the thumb.

## 7. Conclusions

We developed a tactile interaction sensor with four pressure sensor units and three shear sensor units integrated within a 3 × 3 mm area on a single chip. The sensing range and sensitivity of the sensor may be controlled by adjustment of the component dimensions and the arrangement of spatially digitized contact electrodes, resulting in an integrated sensor with low noise, high reliability, and high uniformity. Our pressure sensors possess high sensitivity, enough to detect an ~50 mgf light touch. Our shear sensors show the ability to discriminate shear direction and detect whether there is additional pressure. We demonstrated that the TIS can detect various contact shape and motion. With further developments, the TIS may be applicable to future robotic fingers and remote input interfaces.

## Data Availability

Not applicable.

## Supplementary Materials

The following are available online at https://www.mdpi.com/article/10.3390/s21134274/s1, Figure S1: Explanatory diagram of the screen composition of video clip, Video S1: Slip detection, Video S2: Clockwise motion detection, Video S3: counter-clockwise motion detection.
